# Emerging Biomarkers of Acute Myocardial Infarction, An Overview of the Newest MicroRNAs

**DOI:** 10.31661/gmj.v12i.2909

**Published:** 2023-05-30

**Authors:** Venus Shahabi Raberi, Elnaz Javanshir, Mohsen Abbasnezhad, Sina Mashayekhi, Amirreza Abbasnezhad, Masumeh Ahmadzadeh, Akram Shariati

**Affiliations:** ^1^ Seyed-Al-Shohada Cardiology Hospital, Urmia University of Medical Sciences, Urmia, Iran; ^2^ Cardiovascular Research Center, Tabriz University of Medical Sciences, Tabriz, Iran; ^3^ Faculty of Medicine, Tehran University of Medical Sciences, Tehran, Iran; ^4^ Department of Cardiology, School of Medicine, Urmia University of Medical Sciences, Urmia, Iran

**Keywords:** Myocardial, Biomarker, LcnRNA

## Abstract

Globally, acute myocardial infarction (AMI) is the leading cause of death. Early and precise diagnosis is essential for medical care to enhance prognoses and reduce mortality. The diagnosis of AMI relies primarily on conventional circulating biomarkers. However, these markers have many drawbacks. Non-coding RNAs (ncRNAs) form a significant fraction of the transcriptome and have been shown to be essential for many biological processes, including the pathogenesis of the disease. ncRNAs can be utilized as biomarkers due to their important role in the disease’s development. The current manuscript describes recent progress on the role of ncRNAs as new AMI biomarkers.

## Introduction

Myocardial Infarction (MI) and Its Markers

It is now widely recognized that cardiovascular diseases (CVD) and circulatory diseases are the main causes of mortality worldwide [1][2]. Cerebrovascular and ischemic heart disease (IHD) accounted for most of these CVD deaths [3][4]. One of the primary causes of hospital admission and mortality is acute myocardial infarction (AMI).

It’s critical to correctly determine whether a person with severe chest pain is undergoing an AMI [5]. Suitable markers of AMI should first be quantitatively modified to be used to predict AMI and monitor its pathogenic processes [6]. Cardiac troponins (cTns) are the most frequently used markers in clinical practice for AMI diagnosis. The preferred biomarkers have been cTns T and I (cTnT and cTnI) due to their sensitivity and cardiac selectivity. However, acute myocardial infarction is only one of the conditions that can result in elevated cardiac troponin levels. Therefore, finding specific and sensitive biomarkers for incredibly early AMI diagnosis is crucial [7][8].

Non-coding RNAs

Non-coding RNAs (ncRNAs) are transcribed from the genome but do not encode proteins. ncRNAs have important activities including regulation of gene expression [9]. ncRNAs, including long ncRNAs (lncRNAs), recently identified circular RNAs (circRNAs), small interfering RNAs (siRNAs), and microRNAs (miRNAs), have been demonstrated to have diagnostic and regulatory effects in cardiovascular disorders [10]. MicroRNAs are single-stranded ncRNAs with 21-23 nucleotides that regulate the post-transcriptional process and RNA silencing [11]. NcRNA molecules consisting of 200 or more nucleotides belong to the lncRNAs group. Through interactions with proteins and nucleic acids, lncRNAs control the expression of genes during post-translational, translational, RNA processing, and transcriptional stages. They control lifespan pathways by regulating senescence, apoptosis, differentiation, and cell proliferation [12]. Similar to miRNAs, siRNAs are double-stranded ncRNAs that control genes. They are typically 20-24 (commonly 21) base pairs in length. This genome regulation occurs at critical levels of genome function, including translation, transcription, chromosome segregation, RNA stability, chromatin structure, and RNA processing [13]. CircRNAs have a covalent loop structure and belong to a family of endogenous RNAs. Circular RNAs are less common than other types of RNA, but are very stable due to their circular structure. circRNAs also exhibit a high level of tissue specificity [14].

NcRNAs are expressed in many human tissues, and their expression is particularly tissue-specific in certain circumstances. Due to their unique characteristics, they can be employed as potential biomarkers for the diagnosis of various pathologic diseases, including cancer, myocardial infarction (MI), and other cardiovascular problems [10].

None-coding RNAs and Cardiovascular Disorders

NcRNAs perform a variety of cell- or tissue-specific biological and pathological functions. Additionally, ncRNAs can be used as biomarkers for the detection of cardiovascular disorders due to the presence of ncRNAs in circulatory systems [15]. Researchers have suggested that ncRNAs, especially lncRNAs and microRNAs, play an important role in regulating cardiovascular aging and heart development. MicroRNAs have received the most attention among them due to their role in MI and cardiovascular disorders. Additionally, many lncRNAs were found to be regulated during AMI in cardiac tissue [16]. In the current manuscript, we discussed the most recent advances regarding ncRNAs’ role as new AMI biomarkers.

Search Strategy

Data were collected using the keywords "microRNA" or "miR" or "myocardial infarction" or "biomarker" or "LncRNA" or "ncRNAs" or "Heart" or "MI" or "ncRNA" in the Web of Science, Scopus, and PubMed databases. The title and abstract of all articles were reviewed, and papers related to the objective of our investigation were selected.

MiR-499

The heart contains over 200 miRNAs, and miR-499 is one of the most extensively researched. Exosomes from infarcted mouse hearts were found to release miR-499 into circulation, and their circulating levels were all elevated [17]. MiR-499 level can be utilized to diagnose AMI earlier than traditional markers. While creatine phosphokinase-MB (CK-MB) and cTnI are detectable 2 hours after the onset of chest pain, miR-499 is present in the plasma only 1 hour after the AMI and continues to rise 9 hours later [18]. According to previous studies, the concentration of circulating miR-499 in a cohort of healthy controls was hardly detectable and extremely close to the assay’s detection limit. However, there was an increase in troponin-negative patients in certain patients with non-AMI heart disease including angina pectoris and myocarditis. These findings imply that miR-499 can be used as a possible biomarker to identify early-stage myocarditis and angina pectoris in other diseases [18][19]. In another study, miR-499 showed an increase in patients with acute non-ST-elevation myocardial infarction (NSTEMI) compared to controls in 92 elderly people with NSTEMI, 81 non-AMI patients with chronic heart failure (CHF), and 99 controls. Findings demonstrated that circulating miR-499 is a reliable biomarker of acute NSTEMI, with a diagnostic accuracy greater than cTnT in elderly patients [20].

Before using miR-499 as a reliable biomarker of AMI diagnosis, several issues including the inability to quickly identify miR-499 must be resolved. However, miR-499 has distinct advantages over cTnT and other traditional markers, such as the fact that it has a high level of in vitro stability and is unaffected by renal function. Additionally, giant troponin, troponin antibodies, and other specimen variables can interfere with currently used immunological approaches to detect cTnT and CK-MB. As a result, miR-499 remains a reliable indicator of myocardial damage [18].

MiR-1

MiR-1 is a highly expressed conserved miRNA in the heart and has important implications for cardiac tissue development. MiR-1 has been found to target the transcription factor Heart, and Neural Crest Derivatives Expressed 2 (HAND2), which promotes the development of ventricular cardiomyocytes [21]. Several studies have reported changes in miR-1 during myocardial disorders. In a recent study, the potential of using miR-1 as a substitute for cardiac steatosis biomarkers was explored. Regardless of confounding variables, circulating miR-1 levels were strongly associated with myocardial steatosis. It has been proposed that circulating miR-1 can predict myocardial steatosis on its own. This result highlights the significance of circulating miR-1 as asymptomatic diabetic cardiomyopathy diagnostic tool [22].

Several other studies have reported the diagnostic value of miR-1 in MI. According to research by Enrica Pinchi et al., a reduction in the miR-1 level in blood samples from patients with AMI can be utilized as a biomarker to identify sudden cardiac death (SCD) caused by AMI.

Along with miR-499, the miR-1 demonstrated significant accuracy in separating SCD from AMI. Overexpression of miR-1 has been reported as a potential marker for AMI by downregulating the urothelial carcinoma-associated 1 (UCA1) [23].

UCA1

A lncRNA known as urothelial carcinoma-associated 1 (UCA1) may be useful as a MI diagnostic marker. UCA1 may be used as AMI because it is only expressed in the spleen and heart after birth [24]. In a study, the level of UCA1 in the plasma of patients with AMI and healthy controls was measured to verify this hypothesis. Because it is thought that miR-1 controls the expression of UCA1, they also checked the amount of miR-1. The UCA1 was dropped early but elevated after MI. These results suggest that UCA1 may serve as a possible new marker for AMI detection [25].

The miR-1 gene controls the amount of UCA1. In bladder cancer cells, miR-1 was found to have an Ago2-slicer-dependent effect on decreasing UCA1 expression. The effector RNA-induced silencing complex (RISC), which comprises an Argonaute protein (AGOs 1-4 in humans), mediates small RNA silencing. Plasma from rats or patients with AMI has been reported to have significantly increased amounts of miR-1. The research also revealed a negative correlation between the expression of miR-1 and the UCA1. The results suggest that miR-1/UCA1 axis in circulation may be a more useful predictive and diagnostic marker for AMI compared to miR-1 levels alone [26].

H19

LncRNA H19 (H19), located on chromosome 11p15.5 and encoded by the H19 gene, was one of the first discovered lncRNAs. After transcription and polyadenylation, it is transported from the nucleus to the cytoplasm. This LncRNA is typically expressed in fetal tissues, and after delivery, its expression is significantly diminished. Recently, it was discovered that H19 participates in several pathological processes, including fibrosis progression, neurogenesis, angiogenesis, and inflammatory responses [27].

It is not surprising that the alteration in LncRNA H19 expression occurrs preferentially in heart tissue and is associated with CVD [28][29]. Additionally, cardiac cells become leaky during cardiac muscle injury and release their contents into the bloodstream [30]. A significant increase in circulating H19 transcript levels has been reported in patients with AMI. There was also a direct association between the plasma homocystine and relative H19 expression. To differentiate MI patients from controls, the relative expression of H19 demonstrated 70% sensitivity and 94% specificity. This study also found that the H19 level could be used as a marker for MI; however, more research is required to apply this finding more broadly [31]. In a recent study, the lncRNA H19 change was associated with cardiovascular risk variables including cardiac ejection fraction, lipoprotein A, high-density lipoprotein (HDL), and white blood cell counts and negatively correlated with several cardiovascular protective factors. There was a strong correlation between lncRNA H19 and the cardiac biomarkers CK-MB, CK, and cTnT. Consequently, increased expression of H19 can be considered a potential AMI marker [15].

MiR-133a

MiR-133 and miR-1 share the same chromosomal locations for transcription [32]. In particular, miR-133a has an important impact on several malignancies, including hepatocellular carcinoma and breast cancer, as well as heart development and dysfunction [33]. Furthermore, miR-1 and miR-133a are essential for promoting cardiogenesis, heart health, and pathology. By controlling the cardiac action potential, miR-133a and miR-1 also regulate cardiac automaticity and conductance in the heart [32]. High expression of miR-133 enhances cardiac function by increasing the fractional shortening (FS) and left ventricular ejection fraction (LVEF) [34].

According to a study by Liu Peng1 et al., miR-133 levels were significantly increased in patients with AMI compared to non-MI controls. The miR-133 specificity and sensitivity were 91.2% and 81.1%, respectively [35].

In a study, patients with unstable angina pectoris or acute myocardial infarction have significantly higher serum levels of miR-133a than healthy individuals [36]. MiR-133a serum levels had a strong correlation with all-cause mortality in ACS patients [37]. Kimura et al. showed that when cTnT and CPK serum levels are normal, circulating levels of miR-133a and miR-1 increased shortly after AMI. The blood miR-133 level was more sensitive to myocardial damage than the miR-1 level. In addition to traditional markers, miR-133a may also provide prognostic information, possibly much former [38][36].

Other Important ncRNAs

Many ncRNAs have been shown to be associated with MI. MiR-208a, miR-1, miR-133, and miR-499 were the first miRNAs discovered as markers for MI patients [39]. Several genes are differentially expressed in patients with AMI, including potassium voltage-gated channel, KQT-like subfamily, member one opposite strand/antisense transcript 1 (KCNQ1OT1), metastasis-associated lung adenocarcinoma transcript 1 (MALAT1), cyclin-dependent kinase inhibitor 2B antisense RNA 1 (ANRIL), and hypoxia-inducible factor 1A antisense RNA 2 (aHIF). More importantly, ST-elevation myocardial infarction (STEMI) from NSTEMI could be distinguished by KCNQ1OT1, ANRIL, and MALAT1 [40].

Two miRNAs essential for vascular biology, miR-126, and miR-155, were reduced in serum from patients after off-pump coronary artery bypass graft, pointing to a possible controlling role for miR-126 and miR-155 in cardiac surgery [41]. Following a prior study that suggested serum miR-191 and serum miR-26a were reduced in patients with AMI, a subsequent microarray-based investigation also found the same results [42][43]. Microarray assays revealed that miR-320b and miR-125b levels were lower in the patients with AMI, and this decrease was subsequently confirmed in a cohort of 178 Chinese patients with AMI [44]. Some studies have discovered that heart failure, STEMI, and NSTEMI patients’ plasma miR-145 levels were decreased [45]. Studies showed that miRNA-208b, miRNA-34a, miRNA-328, and MiRNA-134 were also linked to heart failure development and increased risk of mortality after AMI [46].

Limitations

The application of ncRNA screening techniques in clinical practice is currently limited by cost and time requirements. Material selection, sample separation, detection, processing methods, and normalization strategies are among the operational criteria yet to be established. Particularly, ncRNA levels can range significantly between various material selections and various isolation techniques. Another restriction is the ncRNA’s lack of specificity as biomarkers. Since the majority of the sample sizes considered in these studies were small, long-term and follow-up investigations are still required for further confirmation of the clinical application of ncRNAs for AMI diagnosis [47].

## Conclusion

In conclusion, ncRNAs, particularly miRNAs, showed many advantages as biomarkers of AMI. Such biomarkers are arguably required to aid healthcare decisions in the diagnosis and prognosis of AMI and facilitate the transition from conventional diagnostic markers to new methods. However, there are still many challenges to overcome before these ncRNAs can be used therapeutically.

## Conflict of Interest

None.
